# UNDERSTANDING INTERGENERATIONALITY: THEORIES, REFLECTIONS, AND EXPERIENCES

**DOI:** 10.1093/geroni/igz038.552

**Published:** 2019-11-08

**Authors:** Stephanie Hatzifilalithis, Amanda M Grenier

**Affiliations:** 1 McMaster University, Ontario, Canada; 2 McMaster University, Health Aging and Society, Ontario, Canada, Canada

## Abstract

Research into different aspects of intergenerationalities continues to develop at a considerable pace for individuals, communities, and society more generally. A number of programs and practices for older people are organized around the presumed benefits of intergenerational interaction between younger and older people, with intergenerational programming operating as a taken-for-granted practice. However, the merits of this approach, the models that inform practice, and the learning that takes place between older and younger people, remain under-theorized. This poster reviews and discusses dominant theoretical frameworks including reflections and experiences from intergenerational learning programs in Canada (e.g. Co-Housing). It documents how the field of intergenerationality is conceptualized and executed in the realms of theory and practice; how models retain age and stage-based assumptions, including the polarizing discourses of ‘decline’ and ‘activity’; and discusses the implications for methodology, application, and outcome measures. By understanding the underlying assumptions utilized in the field of intergenerational learning, this poster makes an important contribution to the theoretical foundations, methods, and approaches, that are required to build more appropriate intergenerational landscapes.

